# Distinct Gut Microbiome Characteristics Associated with Mental Health Symptoms of Healthy Adults

**DOI:** 10.3390/brainsci16040382

**Published:** 2026-03-31

**Authors:** Soon Lee, Christina B. Welch, Karen Zinka, Michael Evans, Hea Jin Park, Valery V. Lozada-Fernandez, Franklin D. West

**Affiliations:** 1 Department of Nutritional Sciences, University of Georgia, Athens, GA 30602, USA; soon.lee@uga.edu; 2 Kelly Products Inc., Covington, GA 30014, USA; cwelch@kellyreg.com (C.B.W.); kzinka@kellyreg.com (K.Z.); mevans@kellyreg.com (M.E.); 3 Department of Animal and Dairy Science, University of Georgia, Athens, GA 30602, USA; westf@uga.edu; 4 Regenerative Bioscience Center, University of Georgia, Athens, GA 30602, USA

**Keywords:** anxiety, depression, stress, sleep problems, gut microbiome, ANCOM-BC, random forest classifier, 16S rRNA gene sequencing, gut microbiota, mental health

## Abstract

**Highlights:**

**What are the main findings?**

- Healthy adults self-reporting stress, anxiety, depression, or sleep problems exhibited distinct gut microbiome communities relative to asymptomatic participants, including significant differences in α-diversity and β-diversity metrics.
- Differential abundance testing (ANCOM-BC) and supervised machine learning (random forest) identified symptom-associated microbial taxa, with partially overlapping features across methods, suggesting candidate taxa that may be associated with symptom groups.

**What are the implications of the main findings?**

- These exploratory findings suggest that self-reported mental health symptoms in healthy adults may be associated with detectable differences in gut microbial composition. The identified taxa represent candidates for further investigation in larger, prospectively designed cohorts.

**Abstract:**

**Background/Objectives:** Mental health conditions, including stress, anxiety, depression, and sleep problems, represent a significant health concern globally. Mounting evidence suggests a link between mental health and the gut microbiome via the gut–brain axis. However, discrepancies in human microbiome data exist due to the heterogeneity in study design and analytical approaches. Thus, this study aimed to explore the gut microbial characteristics associated with self-reported mental health symptoms using multiple analytical methods. **Methods:** A total of 44 healthy adults, defined as individuals without any major chronic medical conditions, were assessed for mental health symptoms using self-reported questionnaire data. To evaluate gut microbial characteristics, stool samples were collected at six time points over 28 days and underwent 16S rRNA gene sequencing. Differential abundance was assessed via ANCOM-BC, and a random forest classifier was implemented to rank features important for the classification of mental health symptoms. Participants who did not report anxiety, stress, depression, or sleep problems served as the reference group for microbiome comparisons. **Results:** The proportion of participants with self-reported mental health symptoms was 11.4% (stress), 27.3% (depression), 31.8% (anxiety), and 15.9% (sleep problems). Participants reporting mental health symptoms showed differences in gut microbiome composition compared to asymptomatic participants, including variation in alpha- and beta-diversity. Differential analysis identified specific taxa with higher or lower relative abundance in participants reporting specific mental health symptoms. Random forest feature ranking identified partially overlapping taxa across methods, suggesting candidate associations warranting further investigation. **Conclusions:** These exploratory findings suggest that self-reported mental health symptoms in otherwise healthy adults are associated with differences in the gut microbiome. The taxa identified in this study represent candidates for validation in larger, independent cohorts.

## 1. Introduction

Mental health conditions continue to be a significant health concern affecting over one billion individuals globally [1]. Anxiety and depression are the most prevalent of mental disorders, and it is estimated that one in four people will experience a mental disorder in their lifetime [2]. Stress and sleep problems are closely related to mental disorders but are conceptually distinct. These may occur as symptoms within diagnosed psychiatric conditions, as prodromal features, or as independent risk factors that increase vulnerability to and drivers that may exacerbate disease progression [3,4,5,6,7]. Unlike anxiety and depressive disorders, which have established diagnostic criteria (e.g., DSM-5), stress and sleep problems as assessed in population studies typically reflect self-reported experiences rather than clinical diagnoses.

While various biological, social, and psychological factors may contribute to the risk of mental health issues [8,9], rapid advancements in technology, economic pressures, and lifestyle changes have further exacerbated these challenges in modern society. The age-standardized prevalence rates of mental disorders are predicted to increase consistently through 2050 [10]. Mental disorders impose profound effects on patients and their families. Severe cases are associated with shorter life expectancy and suicide [1], underscoring the medical and financial burden for the public. Currently, psychological and pharmacological drugs are common treatment options [11]. However, these interventions are often discontinued due to adverse effects or limited effectiveness [11,12], highlighting the need for a deeper understanding of the biological factors associated with mental health, including the role of the gut microbiome.

In recent years, the association between the gut microbiome and mental health has gained significant interest [13,14]. This involves the bidirectional communication between the gut and the brain through the gut–brain axis, which includes multiple signaling pathways such as microbial short-chain fatty acids (SCFAs), inflammatory cytokines, neuroendocrine signaling, and neurotransmitter production [15]. These processes are ultimately driven by the composition and structure of the gut microbial community. 16S rRNA gene sequencing enables characterization of this community at the taxonomic level, capturing shifts in microbial diversity, richness, and the relative abundance of specific taxa [16]. Dysregulation of these pathways has been associated with stress reactivity [17], circadian disturbance [18], and abnormal mood regulation [15], which are closely related to mental health outcomes. However, human microbiome studies are highly challenging to reproduce, and discrepancies in findings remain a major issue [19], due to various compounding factors such as study design, analytical objectives, technical approaches, and reporting [19,20]. This underscores the need for multiple analytical methods to generate more robust data to better understand the association of the gut microbiome with mental health.

By using multiple analytical approaches, including ANCOM-BC and a Random Forest classifier, this study aimed to contribute to the growing body of literature exploring the microbial characteristics in individuals with mental health symptoms, including stress, anxiety, depression, and sleep problems. Our findings may contribute to the identification of candidate microbial taxa associated with self-reported mental health symptoms, which could inform hypothesis generation for future mechanistic and interventional studies.

## 2. Materials and Methods

### 2.1. Study Participants

Forty-four adults (≥18 years) of both sexes were recruited by Kelly Products Inc. (Covington, GA, USA) and enrolled following informed consent. Enrollment eligibility was defined by the absence of major chronic medical conditions (e.g., diabetes, cancer, cardiovascular disease, specific allergies), absence of use of antibiotics, anti-inflammatory, or gastrointestinal medications within the previous two months, and a body mass index (BMI) between 18.5 and 29.9 kg/m^2^. Exclusion criteria included pregnancy. This definition does not imply the absence of all health concerns, including subclinical or self-perceived mental health symptoms, which were variables of interest. Participants attended an enrollment visit with a trained research staff member for training in the proper use of the Automated Self-Administered 24 h Dietary Assessment Tool (ASA24; National Cancer Institute, Bethesda, MD, USA) and sample collection kits. The study was conducted in compliance with ethical guidelines, and all procedures were approved by the Sterling Institutional Review Board (#11402).

### 2.2. Participant Health and Psychosocial Assessment

Participant health, psychosocial status, and relevant covariates were assessed using a structured, self-administered questionnaire. Psychosocial and sleep-related variables were assessed through direct self-report items embedded within the medical history section, including binary (yes/no) responses indicating a history or current presence of stress, anxiety, depression, and sleep problems. Dietary intake was assessed using ASA24, which was filled out by participants for 12 days (4 weekend days and 8 nonconsecutive weekdays) on days 1, 3, 5, 7, 10, 12, 14, 17, 19, 21, 24, and 26.

### 2.3. Stool Sample Collection and Microbiome Profiling

Stool samples were self-collected at six regular intervals over 28 days using OMNIgene-GUT kits (DNA Genotek, Ottawa, ON, Canada). The collected samples were kept at room temperature until they were returned to KPI headquarters for storage at −80 °C. DNA was extracted from fecal samples following the protocol described in Welch et al. [21] with the following modifications. We used 350 mg of sample placed in 2 mL Lysing Matrix E tubes (MP Biomedicals LLC, Irvine, CA, USA), which were homogenized for 10 min at full speed using QIAGEN vortex adapter (QIAGEN, Venlo, The Netherlands) to disrupt the cells. Enzymatic inhibition was achieved using InhibitEX Buffer (QIAGEN, Venlo, The Netherlands), and DNA elution and purification were carried out using a spin column and a series of specialized buffers according to the manufacturer’s specifications (QIAamp Fast DNA Stool Mini Kit; QIAGEN, Venlo, The Netherlands). DNA concentration and purity in the resulting eluate were assessed fluorometrically using the Qubit 4 Fluorometer (Thermo Fischer Scientific; Waltham, MA, USA). Samples with a minimum volume of 100 μL and 10 ng/μL of DNA were stored at −20 °C until further analysis.

### 2.4. 16S rRNA Gene Sequencing

Following DNA extraction, library preparation and 16S ribosomal ribonucleic acid (rRNA) gene sequencing were performed. The library preparation step included polymerase chain reaction (PCR) amplification using the forward primer S-D-Bact-0341-b-S-17 (5′-CCTACGGGNGGCWGCAG-3′) and reverse primer S-D-Bact-0785-a-A-21 (5′-GACTACHVGGGTATCTAATCC-3′) [22] followed by a PCR clean-up using AMPure XP beads (Beckman Coulter Life Sciences, Indianapolis, IN, USA). A second PCR was performed to attach Illumina’s indices and sequencing adapters (Nextera XT Index Kit; Illumina Inc., San Diego, CA, USA), followed by another PCR clean-up step using AMPure XP beads. Finally, the library was quantified using the Qubit 4 Fluorometer (Thermo Fischer Scientific; Waltham, MA, USA). Paired-end sequencing was performed on the Illumina MiSeq instrument using a 2 × 300 bp configuration (MiSeq v3 reagent kit; Illumina Inc., San Diego, CA, USA) targeting the V3-V4 hypervariable region of the 16S rRNA gene. A well-characterized bacteriophage PhiX genome (PhiX Control v3 Library; Illumina Inc., San Diego, CA, USA) was used as a control for the sequencing runs.

### 2.5. Bioinformatics Analysis

Sequencing analysis was done using QIIME2 version 2024.5 [23]. Raw reads were demultiplexed and quality-filtered using the q2-demux plugin, followed by denoising using DADA2 [24] implemented within QIIME2. Due to the low quality of the reverse reads, we only analyzed forward reads. No trimming was implemented, and we used all the default parameters. All amplicon sequence variants (ASVs) were aligned with MAFFT [25] via the q2-alignment plugin and then used to construct a phylogeny using fasttree2 [26] via the q2-phylogeny plugin. α-diversity metrics (Shannon index, observed features, and Faith’s Phylogenetic Diversity) [26,27] and β-diversity metrics (weighted UniFrac [28], unweighted UniFrac [29], and Bray–Curtis dissimilarity) were estimated using the q2-diversity plugin. Taxonomy was assigned to ASVs using the q2-feature-classifier plugin [30], classify-sklearn naïve Bayes taxonomy classifier against the green genes 2 database (version 2022.10) [31]. Differential abundance analysis was performed using the ANCOM-BC2 package (version 2.12.0) implemented in R (version 4.5.1) [32]. The model included the mental health symptom as the primary fixed effect, with sex, age, and BMI included as covariates. Multiple testing correction was applied using the Benjamini–Hochberg false discovery rate (FDR) procedure, and results are reported as adjusted q-values. Taxa with q < 0.05 were considered differentially abundant. Analyses were conducted using a prevalence filter of 10% (prv_cut = 0.10) and no library size filtering (lib_cut = 0), with pseudo-count sensitivity analysis enabled.

We implemented a subject-level nested cross-validation framework to evaluate the predictive performance of gut microbiome taxa using a Random Forest classifier in Python (v 3.9.19), with data processing performed in pandas (v2.2.2) and NumPy (v1.26.4). The input feature table (taxa count table) was parsed to retain only microbial taxa features. The outcome variable (e.g., Anxiety, Stress, Depression, or Sleep problems) was encoded as a binary categorical variable. To prevent data leakage arising from repeated measures within participants, internal validation of the random forest classifier employed grouped k-fold cross-validation (k = 5), with participant ID as the grouping variable using GroupKFold (scikit-learn v1.4.2). This ensured that all samples from a given participant were assigned exclusively to either the training or test fold within each iteration. Model development included hyperparameter tuning via RandomizedSearchCV within an inner GroupKFold. The RandomForestClassifier was optimized over standard parameters (e.g., number of trees, tree depth, and feature subsampling). Classification performance was assessed using the mean area under the receiver operating characteristic curve (AUC), sensitivity, and specificity across folds. Given the small sample size (*n* = 44 participants), the random forest analysis is presented as an exploratory feature-ranking exercise rather than as evidence of a validated classifier. The trained Random Forest classifiers were externally validated using an independent cohort (*n* = 108) to assess generalizability. Predicted class labels and class probabilities were generated for each participant, and performance was evaluated using accuracy, sensitivity, specificity, and AUC.

### 2.6. Statistics

For α-diversity measurements, we aggregated the values at the participant level across time points and performed Kruskal–Wallis. Significance was assigned as * *p* < 0.05.

For β-diversity, we performed a stratified PERMANOVA using the adonis2 function implemented in the vegan R package. The outcome of interest was specified as the main predictor in the model, and statistical significance was assessed using permutation testing (*n* = 999) constrained with the participant identifier specified as the stratification variable.

## 3. Results

### 3.1. Descriptive Statistics

A total of 44 healthy participants with a mean age of 38.55 (SD 15.7) years were recruited. The characteristics of the participants are shown in Table 1.

No significant differences between sexes were found in terms of age (*p* = 0.9810; Unpaired *t*-test (two-tailed) with Welch’s correction) and BMI (*p* = 0.6390; Unpaired *t*-test (two-tailed) with Welch’s correction; Figure S1). Approximately 79.5% of the participants were female, and 81.9% were white. The proportion of participants with self-reported mental symptoms was 11.4%, 27.3%, 31.8% and 15.9%, for stress, depression, anxiety, and sleep problems, respectively. The intake of macronutrients, fiber, choline, and sodium was assessed due to their known interplay with the gut microbiome [33,34]. The daily intake of nutrients was comparable to the U.S. adult averages.

### 3.2. Participants with Self-Reported Mental Health Symptoms Show Significant Changes in Alpha Diversity

The gut microbiome of participants was analyzed to determine differences in α-diversity across self-reported mental health symptoms (Table 2). Participants with self-reported stress exhibited changes in α-diversity across all three metrics assessed, with decreased Shannon’s index (*p* = 0.009; Kruskal–Wallis), observed features (*p* = 0.058; Kruskal–Wallis), and Faith PD (*p* = 0.047; Kruskal–Wallis). Participants with anxiety had lower α-diversity, shown by a significant reduction in Shannon’s index (*p* = 0.0470; Kruskal–Wallis) and a lower number of observed features (*p* = 0.05; Kruskal–Wallis) compared to those without self-reported anxiety. In contrast, participants with depression and sleep problems did not show significant differences in Shannon’s index, observed features, and Faith PD.

**Figure 1:**
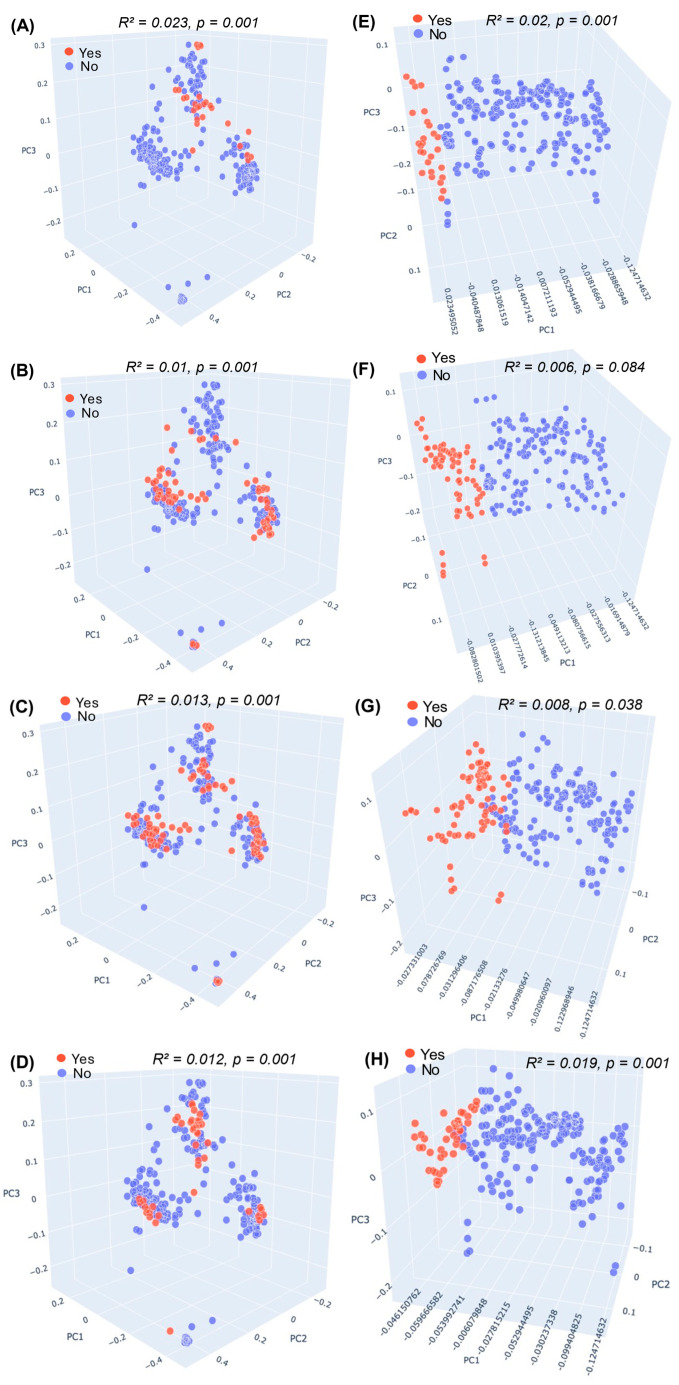
Significant changes in gut microbiota composition between participants with and without self-reported mental health symptoms. PCoA plots of Bray–Curtis distances illustrate significant differences in the gut microbiome of participants with and without self-reported (**A**) stress, (**B**) depression, (**C**) anxiety, and (**D**) sleep problems. PCoA plots of Weighted UniFrac distances illustrate differences in the gut microbiome of participants with and without self-reported (**E**) stress, (**F**) depression, (**G**) anxiety, and (**H**) sleep problems at days 0, 3, 7, 14, 21, and 28. Samples colored red correspond to participants with self-reported conditions, whereas samples colored in blue correspond to normal participants. R^2^ and *p* values shown are for stratified PERMANOVA.

To assess β-diversity, we calculated Bray–Curtis and weighted UniFrac distance matrices and observed that participants reporting mental health symptoms harbored gut microbial communities that differed from those of participants without symptoms (Figure 1; *p* < 0.05). Using Bray–Curtis distances, β-diversity differed significantly between groups for stress (*p* = 0.001, R^2^ = 0.023), anxiety (*p* = 0.001, R^2^ = 0.01), depression (*p* = 0.001, R^2^ = 0.03), and sleep problems (*p* = 0.002, R^2^ = 0.012) (Figure 1A–D). Using weighted UniFrac distances, between-group differences were significant for stress (*p* = 0.001, R^2^ = 0.02), anxiety (*p* = 0.038, R^2^ = 0.008), and sleep problems (*p* = 0.001, R^2^ = 0.019), whereas the association for depression did not reach statistical significance (*p* = 0.084, R^2^ = 0.006) (Figure 1E–H).

### 3.3. Participants with Self-Reported Stress Show Significant Microbial Alterations and Distinct Gut Microbiome Features

**Figure 2:**
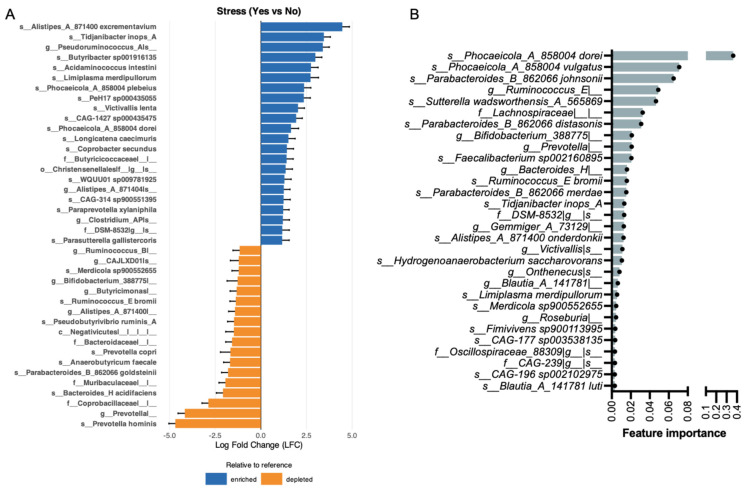
Gut microbiome features associated with stress. (**A**) Differentially abundant taxa identified by ANCOM-BC with Stress as the primary fixed effect and adjustment for sex, age, and BMI. Log fold changes (LFC) represent bias-corrected differences in log abundance (Yes vs. No). Error bars denote Wald 95% confidence intervals (β ± 1.96 × SE). Significance was assessed using Wald tests with Benjamini–Hochberg FDR correction; taxa with q ≤ 0.05 were considered significant. (**B**) Bar plot of the top 30 most important microbial features for classifying participants by stress status. A nested subject-level cross-validated Random Forest model was trained on taxa-level abundance data, and feature importance was computed using mean decrease in Gini. Bars are ordered by decreasing importance, indicating each feature’s relative contribution to classification performance.

ANCOM-BC analysis identified 138 taxa as differentially abundant between participants reporting and not reporting stress (q < 0.05) (Figure 2A shows the top 40). Among the top enriched taxa were *s__Alistipes_A_871400 excrementavium* (logFC = 4.46, q = 5.54 × 10^−19^), *s__Tidjanibacter inops_A* (logFC = 3.45, q = 2.52 × 10^−13^), *g__Pseudoruminococcus_A|s__*(logFC = 3.40, q = 1.13 × 10^−13^). Among the top depleted taxa were *s__Prevotella hominis* (logFC = −4.69, q = 2.01 × 10^−26^), *g__Prevotella|__* (logFC = −4.17, q = 4.27 × 10^−44^), and *f__Coprobacillaceae|__|__* (logFC = −2.88, q = 1.35 × 10^−17^).

To identify gut microbiome features associated with stress, a random forest classifier was applied to taxa-only microbiome features to discriminate stress status (n = 262 samples from 44 subjects). The model achieved an out-of-fold ROC-AUC of 0.672, indicating modest ability to distinguish between participants with and without self-reported stress. Overall accuracy was high (0.882), largely driven by strong specificity (0.970), whereas sensitivity was low (0.172) (Table S1), reflecting limited ability to correctly identify stressed individuals. Balanced accuracy was 0.571, and F1 scores were 0.590 (macro) and 0.859 (weighted), further highlighting class imbalance in predictive performance. Feature importance analysis, based on the mean decrease in Gini impurity, identified a subset of microbial taxa contributing to model discrimination (Figure 2B). The top ranked features were *s__Phocaeicola_A_858004 dorei*, *s__Phocaeicola_A_858004 vulgatus* and *s__Parabacteroides_B_862066 johnsonii.* Overlapping taxa across methods include the species *Phocaeicola A 858004 dorei*, *Limiplasma merdipullorum*, *Tidjanibacter inops_A*, and *Ruminococcus_E bromii* and the genus *Prevotella.*

### 3.4. Participants with Self-Reported Anxiety Display Distinct Microbial Shifts and Anxiety-Related Gut Microbiome-Associated Taxa

**Figure 3:**
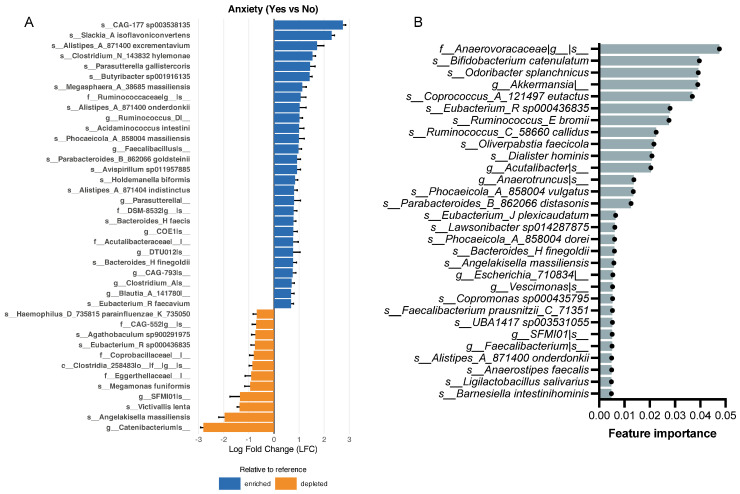
Gut microbiome features associated with anxiety. (**A**) Differentially abundant taxa identified by ANCOM-BC with Anxiety as the primary fixed effect and adjustment for sex, age, and BMI. Log fold changes (LFC) represent bias-corrected differences in log abundance (Yes vs. No). Error bars denote Wald 95% confidence intervals (β ± 1.96 × SE). Significance was assessed using Wald tests with Benjamini–Hochberg FDR correction; taxa with q ≤ 0.05 were considered significant. (**B**) Bar plot showing the top 30 most important features to classify participants by anxiety. A nested subject-level cross-validated Random Forest model was trained on taxa-level abundance data, and feature importance was computed using mean decrease in Gini.

Using the ANCOM-BC method, we identified 181 taxa as differentially abundant between participants reporting and not reporting anxiety (Figure 3A shows the top 40 taxa). Among the top enriched taxa, *s__CAG-177 sp003538135* (logFC = 2.74, q = 3.10 × 10^−30^), *s__Slackia_A isoflavoniconvertens* (logFC = 2.97, q = 9.02 × 10^−25^), and *s__Alistipes_A_871400 excrementavium* (logFC = 1.72, q = 1.79 × 10^−12^) were found. Among the top depleted taxa, *g__Catenibacterium|s__* (logFC = −2.81, q = 1.32 × 10^−26^), *s__Angelakisella massiliensis* (logFC = −1.96, q = 7.83 × 10^−23^), and *s__Victivallis lenta* (logFC = −1.36, q = 4.10 × 10^−18^) were found.

To identify gut microbiome features associated with anxiety, a random forest classifier was applied to taxa-only microbiome features to discriminate anxiety status. The classifier exhibited limited predictive performance, with an out-of-fold ROC-AUC of 0.467. Overall accuracy was 0.611, with a balanced accuracy of 0.479, reflecting poor discrimination between groups. Model performance was characterized by high specificity (0.838) but very low sensitivity (0.120) (Table S1), indicating a strong bias toward correctly identifying non-anxious individuals while failing to detect anxiety cases. A subset of top-ranked microbial taxa contributing to model discrimination included *f__Anaerovoracacea*, *s__Bifidobacterium catenulatum*, and *s__Odoribacter splanchnicus* (Figure 3B). Importantly, several of the taxa identified among the top features for classification overlapped with taxa detected as differentially abundant by ANCOM-BC. The most significant commonality between the groups was the species *Angelakisella massiliensis*, *Eubacterium R sp000436835*, *Bacteroides H finegoldii*, and *Alistipes A 871400 onderdonkii*, and the genus *SFMI01.*

### 3.5. Participants with Self-Reported Depression Exhibit Selective Microbial Changes

**Figure 4:**
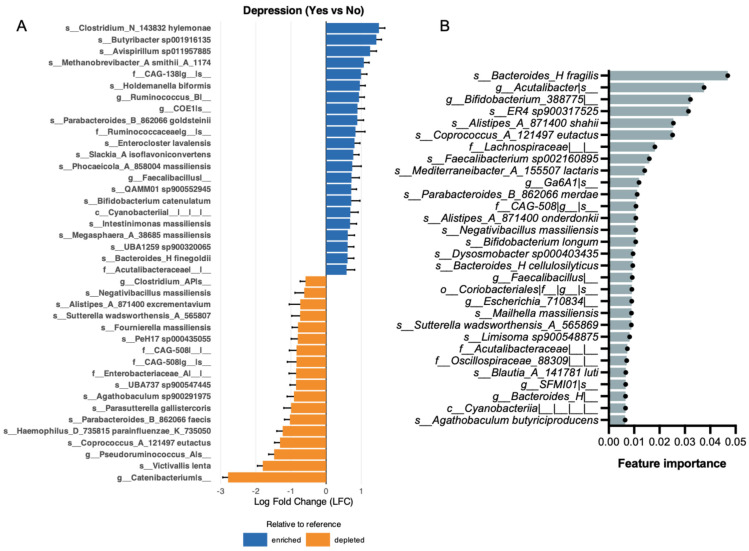
Gut microbiome features associated with depression. (**A**) Differentially abundant taxa identified by ANCOM-BC with Depression as the primary fixed effect and adjustment for sex, age, and BMI. Log fold changes (LFC) represent bias-corrected differences in log abundance (Yes vs. No). Error bars denote Wald 95% confidence intervals (β ± 1.96 × SE). Significance was assessed using Wald tests with Benjamini–Hochberg FDR correction; taxa with q ≤ 0.05 were considered significant. (**B**) Bar plot showing the top 30 features to classify participants by depression. A nested subject-level cross-validated Random Forest model was trained on taxa-level abundance data, and feature importance was computed using mean decrease in Gini.

ANCOM-BC analysis revealed 149 taxa as differentially abundant between participants reporting and not reporting depression (Figure 4A shows the top 40). Among the top enriched taxa, we found *s__Clostridium_N_143832 hylemonae* (logFC = 1.50, q = 7.72 × 10^−13^), *s__Butyribacter sp001916135* (logFC = 1.43, q = 1.83 × 10^−15^), and *s__Avispirillum sp011957885* (logFC = 1.25, q = 1.38 × 10^−11^). Among the top depleted taxa, we found *g__Catenibacterium|s__*(logFC = −2.79, q = 5.65 × 10^−22^), *s__Victivallis lenta* (logFC = −1.80, q = 1.61 × 10^−16^) and *g__Pseudoruminococcus_A|s__* (logFC = −1.48, q = 3.99 × 10^−13^).

To identify gut microbiome features associated with depression, a Random Forest classifier was trained using taxa-only microbiome features (1083 taxa) to classify depression status. The model performed showed poor predictive performance, with an out-of-fold ROC-AUC of 0.394. Overall accuracy was 0.714, but balanced accuracy remained low (0.540), reflecting limited discriminatory capacity. The model exhibited high specificity (0.926) but low sensitivity (0.153) (Table S1), indicating a strong bias toward correctly identifying non-depressed individuals while failing to detect depression cases. Correspondingly, F1 scores were modest (macro F1 = 0.526; weighted F1 = 0.660). Feature importance analysis, derived from the mean decrease in Gini impurity (Figure 4B) shows the top-ranked taxa as *s__Bacteroides_H fragilis*, *g__Acutalibacter*, and *g__Bifidobacterium_388775*. The random forest classifier identified some of the same taxa as ANCOM-BC. The taxa that were consistently identified across both methods were the class Cyanobacteriia, the family CAG-508, the genera *COE1* and *Faecalibacillus*, and the species *Coprococcus A 121497 eutactus* and *Bifidobacterium catenulatum*.

### 3.6. Participants with Self-Reported Sleep Problems Demonstrate Microbial Shifts and Distinct Gut Microbiome Signatures

**Figure 5:**
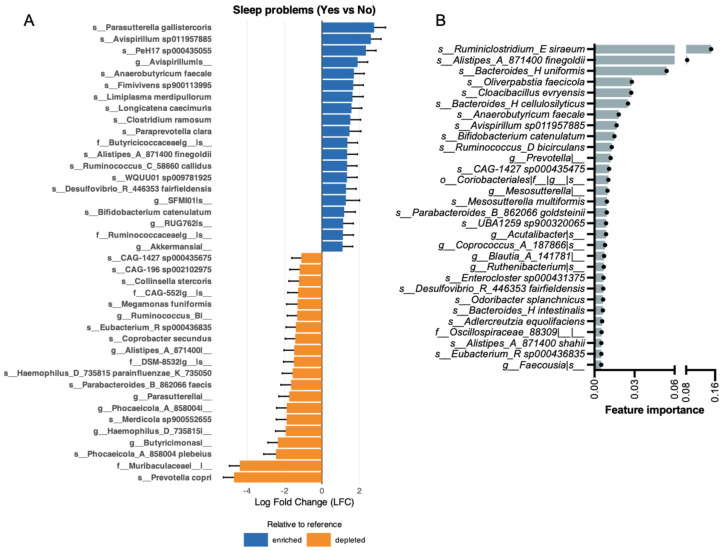
Gut microbiome features associated with sleep problems. (**A**) Differentially abundant taxa identified by ANCOM-BC with sleep problems as the primary fixed effect and adjustment for sex, age, and BMI. Log fold changes (LFC) represent bias-corrected differences in log abundance (Yes vs. No). Error bars denote Wald 95% confidence intervals (β ± 1.96 × SE). Significance was assessed using Wald tests with Benjamini–Hochberg FDR correction; taxa with q ≤ 0.05 were considered significant. (**B**) Bar plot showing the top 30 most important features to classify participants by sleep problems. A nested subject-level cross-validated Random Forest model was trained on taxa-level abundance data, and feature importance was computed using mean decrease in Gini.

ANCOM-BC analysis revealed 67 taxa as differentially abundant between participants reporting and not reporting sleep problems (Figure 5A shows the top 40). Among the top enriched we found *s__Parasutterella gallistercoris* (logFC = 2.79, q = 3.91 × 10^−9^), *s__Avispirillum sp011957885* (logFC = 2.62, q = 5.50 × 10^−8^), and *s__PeH17 sp000435055* (logFC = 2.35, q = 7.52 × 10^−7^). Among the top depleted taxa, we found *s__Prevotella copri* (logFC = −4.71, q = 3.32 × 10^−16^), *f__Muribaculaceae|__|__* (logFC = −4.40, q = 8.88 × 10^−12^), and *s__Phocaeicola_A_858004 plebeius* (logFC = −2.46, q = 2.21 × 10^−9^).

To identify gut microbiome features associated with sleep problems, a random forest classifier was applied. The model demonstrated modest predictive performance, with an out-of-fold ROC-AUC of 0.611. Overall accuracy was 0.782, while balanced accuracy was lower (0.524), reflecting uneven performance across classes. Like other outcomes, the model exhibited high specificity (0.905) but low sensitivity (0.143) (Table S1), indicating strong performance in identifying individuals without sleep problems but poor detection of affected individuals. F1 scores were moderate (macro F1 = 0.524; weighted F1 = 0.762). Feature importance analysis, calculated using the mean decrease in Gini, identified a subset of taxa with relatively greater contributions to classification (Figure 5B). The top-ranked features are *s__Ruminiclostridium_E siraeum*, *s__Alistipes_A_871400 finegoldii*, and *s__Bacteroides_H uniformis.* The random forest classifier identified some of the same top taxa as ANCOM-BC. The top taxa that were consistently identified across methods were the species *Alistipes A 871400 finegoldii*, *Anaerobutyricum faecale*, *Avispirillum sp011957885*, *Bifidobacterium catenulatum*, *Desulfovibrio R 446353 fairfieldensis*, and *Eubacterium R sp000436835.*

### 3.7. External Validation of Random Forest Classifiers Using an Independent Cohort

The trained Random Forest classifiers were externally validated using an independent cohort to assess generalizability. The validation cohort consisted of 108 independent subjects (Table S2). Contrary to the study cohort, the validation cohort included obese individuals and subjects with chronic diseases, as these were not excluded.

In the independent validation cohort, classifier performance demonstrated high specificity but markedly reduced sensitivity across all outcomes, indicating limited ability to correctly identify symptomatic individuals (Table S3). The stress classifier achieved an overall accuracy of 0.75, with specificity (0.769) but low sensitivity (0.30), suggesting that most non-stress participants were correctly classified, while most stress cases were missed. A similar pattern was observed for anxiety (accuracy = 0.731; specificity = 1; sensitivity = 0) and depression (accuracy = 0.815; specificity = 1; sensitivity = 0). The sleep problems classifier showed accuracy of 0.852, with specificity of 1 and sensitivity of 0. Collectively, these findings indicate that, although the models retained strong performance in identifying non-symptomatic individuals, they demonstrated poor sensitivity and limited generalizability for detecting symptomatic participants in the external validation cohort.

## 4. Discussion

The current study aimed to determine the distinct gut microbiome characteristics of participants with self-reported mental health symptoms, including stress, anxiety, depression, and sleep problems, in a cohort of 44 adults. By integrating compositional differential abundance testing (ANCOM-BC) with supervised machine learning (Random Forest classifier), we identified candidate microbial taxa whose abundance differed between symptomatic and asymptomatic participants. Our findings reinforce the concept of a gut–brain axis in mental health and suggest that even subclinical, self-perceived symptoms in otherwise healthy individuals are linked to measurable shifts in gut microbial community structure and composition. These observations align with contemporary models of the microbiota–gut–brain axis, which emphasize the complex integration of neural, immune, endocrine, and metabolic pathways in mediating behavioral and psychological outcomes [35].

Across all symptom categories, we observed significant shifts in overall microbial community structure (β-diversity, Figure 1) supporting the hypothesis that even subclinical psychological symptoms are associated with measurable gut microbial alterations. Although the PERMANOVA R^2^ values were modest, small effect sizes are common in multifactorial traits within heterogeneous human populations and do not preclude biological relevance [19]. These findings align with established models of bidirectional gut–brain communication [15]. Notably, reductions in alpha diversity were observed in participants reporting stress (Shannon index and Faith’s PD) and anxiety (Shannon index) (Table 2), consistent with prior studies linking reduced microbial diversity to depression [14,36], anxiety [12,36], stress [37], and sleep problems [38,39]. Interestingly, results in the literature remain inconsistent [15]. Notably, a systematic review reported that most studies do not identify significant links between α- and β-diversity measures and psychological stress [37]. Moreover, while alterations in microbial composition and diversity are highly relevant to mental health conditions, a decrease in diversity may not necessarily translate to negative outcomes, especially if beneficial taxa are increased [15,40]. Further studies are required to clarify the role of microbial diversity and its reliability as a biomarker for mental health conditions.

Differential abundance analysis using ANCOM-BC revealed several taxa became enriched or depleted across symptom groups (Figure 2, Figure 3, Figure 4 and Figure 5), many of which converged on shared functional themes. Several depleted taxa across anxiety, depression, and stress belong to the well-characterized short-chain fatty acid (SCFA)-producing clades, including *Coprococcus*, *Eubacterium*, and *Faecalibacterium*. Decreased levels of microbiota-derived butyrate have been implicated in the pathogenesis of neuropsychological conditions [41,42]. Butyrate can enter systemic circulation and cross the blood–brain barrier to act directly on the brain or indirectly signal through the vagus nerve to maintain brain-derived neurotrophic factor levels [43,44] potentially improving depressive symptoms [17]. Reduced butyrate levels in individuals with depression are frequently reported [41], and its supplementation has demonstrated anti-inflammatory and neuroprotective effects [45]. In the gut, butyrate facilitates intestinal barrier integrity by upregulating tight junction proteins and reducing inflammation, thereby preventing pathogen infiltration [46]. Our findings on the alterations of butyrate-producing taxa are consistent with the hypothesis that microbial metabolites such as butyrate may contribute to gut–brain signaling relevant to symptom expression. However, because sequencing-based taxonomic inference does not directly measure metabolite production, functional validation is required to substantiate this mechanism.

Implementation of random forest classifiers identified gut microbiome features associated with each mental health symptom (Figure 2, Figure 3, Figure 4 and Figure 5). The use of complementary analytical approaches revealed specific taxa that consistently emerged as top features linked to each symptom group. Together, our results demonstrate distinct alterations in the abundance and diversity of gut microbiota in participants with mental health symptoms, underscoring the potential importance of gut microbiota in mental health.

Among taxa depleted in participants with anxiety, *Eubacterium R sp000436835* is consistent with prior reports linking the *Eubacterium* genus to anxiety and depression, as members of this genus are well-characterized SCFA producers whose reduction may compromise gut barrier integrity and neuroimmune signaling [47,48,49]. *Alistipes A 871400 onderdonkii*, identified by both methods, belongs to a genus increasingly implicated in gut–brain axis signaling [50]. Although *Alistipes onderdonkii* itself is a relatively recently described species [51], its specific role in anxiety warrants further investigation. *Angelakisella massiliensis* was among the top depleted taxa by ANCOM-BC and was independently identified by the random forest classifier, yet its role in the gut–brain axis has not been previously reported, representing a novel candidate for future investigation. *Victivallis lenta*, a syntrophic hydrogen-producing anaerobe [52], was similarly depleted in both anxiety and depression groups, although its functional relevance to the gut–brain axis remains poorly understood. Among enriched taxa, *Slackia A isoflavoniconvertens*, a species involved in polyphenol and isoflavone metabolism [53], is of interest given emerging links between microbial polyphenol metabolism and neuroprotective signaling [54], though its direct relevance to anxiety has not been established. *Odoribacter splanchnicus*, a top-ranked random forest feature, is a known butyrate producer whose depletion has been associated with inflammatory bowel conditions [55], suggesting a plausible though unconfirmed link to anxiety via metabolite-mediated pathways. Collectively, the anxiety-associated taxa identified in our cohort converge on a pattern of depleted SCFA-producing and fiber-fermenting microbes, consistent with the hypothesis that reduced microbial metabolite production may contribute to disrupted gut–brain signaling in anxiety. Given the modest discriminatory performance of the random forest classifier, however, these associations should be interpreted as hypothesis-generating and require validation in larger, clinically characterized cohorts.

The identification of *Coprococcus A 121497 eutactus* as an overlapping taxon across both methods is consistent with one of the most replicated findings in gut–brain axis research. *Coprococcus* species are prominent butyrate producers, and their depletion has been linked to depression in multiple large-scale cohorts [41,42]. Reduced butyrate availability may impair intestinal barrier function and promote systemic inflammation, both of which are implicated in the pathophysiology of depression [41,45]. *Bacteroides H fragilis*, the top-ranked random forest feature, has been linked to depression via multiple proposed mechanisms, including tryptophan metabolism, modulation of inflammatory signaling, and GABA production [56]; however, given considerable strain-level functional heterogeneity, its role may be context-dependent. *Bifidobacterium 388775*, also a top-ranked random forest feature, belongs to a genus with well-established beneficial roles in gut homeostasis. Its depletion has been implicated in the pathogenesis of depression [57], consistent with its functions in acetate production, mucosal barrier maintenance, and cross-feeding interactions with butyrate-producing bacteria [58,59,60]. The depletion of *Catenibacterium* and *Victivallis lenta* in depressed participants mirrors findings from the anxiety group, suggesting shared microbial signatures across these comorbid conditions. *Victivallis lenta* is a syntrophic, hydrogen-producing anaerobe [48] whose functional role in gut–brain communication has not been well characterized, representing a candidate for further investigation. The enrichment of *Butyribacter sp001916135* in depressed participants is notable given that this genus is typically associated with butyrate production. Its enrichment may reflect compensatory shifts in the microbial community in response to the depletion of other SCFA producers, or it may indicate context-dependent functional roles that differ from genus-level assumptions. *Clostridium N 143832 hylemonae*, the most significantly enriched species by ANCOM-BC, belongs to a genus with diverse metabolic capabilities, including bile acid transformation [61] which may influence the gut–brain axis via bile acid receptor signaling [62]. However, the specific functional contribution of this species to depressive symptomatology remains unexplored.

Stress-associated alterations overlapped partially with depression- and anxiety-related taxa, which is not surprising since stress and mental disorders have a compounding effect on one another [3,5]. The most prominent finding was the marked depletion of *Prevotella* and *Prevotella hominis*, ranking among the most significantly altered taxa across all symptom categories in our study. *Prevotella* is a key fiber-fermenting genus associated with plant-rich diets and SCFA production, particularly propionate and succinate [63]. Its depletion in stressed participants is consistent with reports linking reduced *Prevotella* abundance to psychological distress and Western dietary patterns [64,65], and may reflect diet–microbiome interactions that are modulated by stress-related behavioral changes. The Coprobacillaceae family was also significantly depleted in stressed participants. Members of Coprobacillaceae, including *Coprococcus* species, are well-characterized butyrate producers whose depletion has been consistently associated with depression and anxiety [42,43,44], reinforcing the convergence of stress- and mood-related microbial alterations on SCFA-producing taxa. Among the most significantly enriched taxa were *Alistipes A 871400 excrementavium* and *Tidjanibacter inops A*. The genus *Alistipes* has been increasingly implicated in gut–brain axis signaling [50] with certain species linked to stress and fatigue conditions. However, *A. excrementavium* itself remains poorly characterized in the context of mental health. *Tidjanibacter inops A*, identified by both ANCOM-BC and as an overlapping taxon with the random forest classifier, is a relatively recently described organism [50] with limited functional annotation, representing a novel candidate warranting further investigation. Feature importance analysis identified *Phocaeicola A dorei* and *Phocaeicola A vulgatus* as the two top-ranked features, both also identified by ANCOM-BC, with *P. dorei* identified as an overlapping taxon across methods. The co-identification of these two closely related species suggests species-specific metabolic divergence within this genus. Existing evidence highlights probiotic effects of *P. dorei*, with healthy individuals displaying higher abundance of this taxon compared to patients with depression [66] and anti-inflammatory roles via the attenuation of lipopolysaccharide production. This is further supported by studies reporting differential metabolic associations, with *P. vulgatus* positively correlated with butyrate synthesis pathway and *P. dorei* negatively correlated with propionate degradation, potentially modulating the gut–brain axis via distinct metabolic and neuro-inflammatory mechanisms [67]. Additional overlapping taxa across both methods included *Limiplasma merdipullorum*, *Ruminococcus E bromii*, and the genus *Prevotella*, further supporting the robustness of the stress-associated microbial signatures identified in this study. Collectively, the stress-associated microbial profile was characterized by a broad depletion of fiber-fermenting and SCFA-producing taxa, coupled with enrichment of taxa whose functional roles in the gut–brain axis are still being elucidated. These findings are consistent with the hypothesis that stress may disrupt microbial metabolite production and gut homeostasis, potentially contributing to the well-documented bidirectional relationship between stress and gastrointestinal function.

Sleep problems were characterized by depletion of *Prevotella copri*, a dominant member of the human gut microbiome associated with plant-rich diets, complex carbohydrate fermentation, and SCFA production [68]. Its pronounced depletion in participants with sleep problems parallels *Prevotella* depletion observed in the stress group and may reflect shared dietary or lifestyle alterations that accompany both conditions. *Muribaculaceae* was also markedly depleted. This family, a prominent mucin-degrading and SCFA-producing clade particularly well characterized in murine models [69], has been linked to circadian disruption and metabolic perturbation in animal studies [70]. However, its relevance in human sleep–microbiome interactions requires further investigation. *Phocaeicola A plebeius* was also significantly depleted, consistent with the broader pattern of reduced fiber-fermenting taxa in participants with sleep problems. Among the most significantly enriched taxa were *Parasutterella gallistercoris* and *Avispirillum sp011957885*. *Parasutterella* has been increasingly recognized as a core member of the human gut microbiome, with potential roles in bile acid metabolism and succinate production [71]. Altered *Parasutterella* abundance has been reported in metabolic and inflammatory conditions [72], although its specific role in sleep regulation has not been established. *Avispirillum sp011957885* was also enriched in the depression group, suggesting potential overlap in sleep- and mood-related microbial features. Feature importance analysis identified *Ruminiclostridium E siraeum*, *Alistipes A 871400 finegoldii*, and *Bacteroides H uniformis* as the top-ranked taxa. Several taxa were consistently identified across both methods, including *Alistipes A finegoldii*, *Anaerobutyricum faecale*, *Avispirillum sp011957885*, *Bifidobacterium catenulatum*, *Desulfovibrio R fairfieldensis*, *and Eubacterium R sp000436835,* lending additional confidence to their association with sleep problems. *Alistipes A finegoldii*, identified by both ANCOM-BC and the random forest classifier, is notable given the frequent observation of altered *Alistipes* levels in sleep–microbiome studies [73] with a recent study reporting increased abundance of *A. finegoldii* in children with low sleep efficiency [74]. Many *Alistipes* spp. are capable of tryptophan metabolism and indole production, potentially influencing serotonin and melatonin pathways central to circadian regulation [75]. Dysregulation of tryptophan metabolism toward the kynurenine pathway during sleep disruption has been associated with neuroinflammatory processes, providing a plausible mechanistic framework. *Ruminiclostridium E siraeum*, the top-ranked random forest feature, has been implicated in mood and sleep regulation in prior studies [76]. *Bacteroides H uniformis*, the third-ranked random forest feature, is a versatile carbohydrate-degrading species with immunomodulatory properties [77]. While *Bacteroides* species often appear to have positive associations with sleep [78], species-level duality has been reported [79,80], suggesting context-dependent effects. The convergence of SCFA-producing taxa (*Anaerobutyricum faecale*, *Eubacterium* R *sp000436835*) among the overlapping features further supports the theme of altered fermentative capacity in participants with sleep problems. Collectively, the sleep-associated microbial profile was characterized by pronounced depletion of fiber-fermenting taxa—most notably *Prevotella* copri and *Muribaculaceae*—coupled with enrichment of taxa involved in tryptophan and bile acid metabolism. These findings are consistent with emerging models linking microbial metabolite production to circadian regulation and sleep quality, although causal directionality cannot be established from the present cross-sectional design.

Random Forest classifiers were applied here as an exploratory feature-ranking tool rather than as validated diagnostic models. Importance scores partially overlapped with ANCOM-BC findings across symptom groups, providing convergent support for candidate taxa. Internal cross-validated performance, estimated using grouped k-fold cross-validation (GroupKFold, k = 5) with participant ID as the grouping variable to prevent within-participant data leakage, was modest across all outcomes. AUC ranged from 0.394 (depression) to 0.672 (stress), and balanced accuracy ranged from 0.479 (anxiety) to 0.571 (stress). Overall accuracy was numerically higher (0.611–0.882) but was driven by strong specificity and consistently low sensitivity, reflecting a strong bias toward the majority non-symptomatic class rather than genuine discriminatory capacity. External validation in an independent cohort (*n* = 108; Table S2) confirmed limited generalizability across all classifiers (Table S3). The stress classifier achieved an accuracy of 0.75 (specificity = 0.769; sensitivity = 0.30), while the anxiety, depression, and sleep classifiers showed high specificity (0.841–1.00) with near-zero sensitivity (0–0.10), indicating that symptomatic individuals were largely undetected in the external cohort. Reduced performance upon external validation is common in microbiome-based machine learning studies and reflects well-documented challenges in model transportability [81,82,83]. Contributing factors include cohort heterogeneity—the validation cohort included obese participants and individuals with chronic diseases excluded from the discovery cohort—as well as class imbalance and symptom prevalence differences between cohorts. Models trained on imbalanced data may implicitly learn prevalence-dependent decision boundaries [84] that deteriorate when applied to populations with different base rates. Collectively, these findings reinforce the interpretation of the random forest results as a hypothesis-generating feature-ranking exercise and highlight the distinction between identifying statistically significant microbial correlates and developing clinically actionable diagnostic tools.

Several limitations warrant consideration. Firstly, the study cohort sample size (*n* = 44) was modest, which may limit the generalizability of findings and the statistical power to detect subtle associations. Secondly, mental health status was assessed via binary self-report rather than validated clinical scales (e.g., PHQ-9, GAD-7), and may not capture symptom severity. Thirdly, while we adjusted for key covariates (sex, age, BMI), residual confounding from diet, lifestyle, supplement use, and sociodemographic factors cannot be excluded. Fourthly, although participant-level cross-validation was used to prevent within-participant data leakage in the random forest analysis, external validation on an independent dataset yielded low sensitivity. Accordingly, the random forest results should be interpreted as an exploratory feature-ranking exercise. Furthermore, taxonomic profiling does not directly capture microbial function or metabolite production, and causal inference cannot be established in this observational framework.

The study also possesses notable strengths. We used ANCOM-BC, which explicitly accounts for the compositional structure of microbiome sequencing data and corrects for sampling fraction bias, yielding unbiased log-fold change estimates and improved false discovery rate control compared with methods based on relative abundances or rarefaction [32,85]. To further strengthen inference, ANCOM-BC results were complemented with a Random Forest classifier, enabling identification of taxa that were statistically significant and highly predictive, thereby increasing robustness and biological interpretability in this high-dimensional dataset [86]. The convergence of findings across both analytical approaches allows for more confident identification of symptom-associated microbial features. Furthermore, this study incorporated multiple time points for gut microbiome sample collection. As the gut microbial community can shift rapidly—often within days—particularly in response to dietary intake [87,88], studies relying on a single time point may inadequately capture the dynamic nature of microbial communities. While the majority of gut microbiome research is limited to a single time point [88], the current study collected fecal samples at six different time points from each individual throughout 28 days.

## 5. Conclusions

Our results demonstrate that self-reported stress, anxiety, depression, and sleep problems are associated with distinct yet partially overlapping alterations in gut microbial composition. These alterations converge on functional themes involving SCFA-production, tryptophan metabolism, and inflammatory signaling, highlighting the role of the gut microbiome within the gut–brain axis to influence mental health. While external validation underscores current limitations in predictive generalizability, the convergence of compositional and machine learning approaches strengthens evidence for symptom-associated microbial signatures. Future investigations should incorporate larger, diverse cohorts and focus on mechanistic validation to better clarify the causality of the gut microbiome in neuropsychiatric health.

## Figures and Tables

**Table 1:** Study participants’ characteristics. Summary of the distribution of sex, age, BMI, race, and self-reported mental health symptoms within the study cohort. Mean (SD) daily dietary intake values are provided for energy, macronutrients, fiber, sodium, and choline. Data reported as mean ± standard deviation.

Variable	
Sex	*n* (%)
Male	9 (20.5)
Female	35 (79.5)
Age, Mean (SD)	38.5 (15.7)
BMI, Mean (SD)	24.3 (2.95)
Race	*n* (%)
White	36 (81.9)
Black	3 (6.8)
Hispanic	3 (6.8)
Asian	2 (4.5)
Mental Health Symptoms	*n* (%)
Stress	5 (11.4)
Depression	12 (27.3)
Anxiety	14 (31.8)
Sleep Problems	7 (15.9)
Dietary Intake	Mean (SD)
Energy (Kcal)	1911.9 (519.3)
Protein (g)	78.3 (25.8)
Total Fat (g)	85.2 (26.7)
Carbohydrates (g)	200.5 (66)
Fiber (g)	17.8 (7.5)
Choline (mg)	328.3 (133)
Sodium (mg)	3238.6 (964.6)

**Table 2:** Associations between self-reported mental health symptoms and gut microbial alpha diversity metrics. Shannon’s Index, observed features, and Faith’s PD are compared between participants with and without self-reported anxiety, depression, stress, and sleep problems. Values are presented as group means, with corresponding *p*-values indicating the significance of between-group differences assessed using the Kruskal–Wallis test.

Symptom		Shannon’s Index	Observed Features	Faith’s PD
Stress	Yes	7.089	252.433	17.44
	No	7.226	319.413	21.281
	*p*	0.009	0.058	0.047
Depression	Yes	7.47	298.375	21.96
	No	7.551	317.922	20.33
	*p*	0.6306	0.415	0.256
Anxiety	Yes	7.372	280.786	19.838
	No	7.667	332.306	21.332
	*p*	0.047	0.05	0.266
Sleep Problems	Yes	7.445	293.68	18.654
	No	7.469	327.33	21.657
	*p*	0.447	0.3507	0.2809

## Data Availability

The data presented in this study are available from the corresponding authors upon reasonable request with appropriate safeguards (e.g., data-use agreement and IRB-compliant conditions). The data are not publicly available because the associated dataset includes human participant questionnaire responses and associated metadata linked to microbiome profiles, collected under IRB oversight.

## Supplementary Materials

The following supporting information can be downloaded at https://www.mdpi.com/article/10.3390/brainsci16040382/s1: Figure S1: No Significant differences between sexes in age and BMI; Table S1: Random forest classification performance using participant-level grouped cross-validation; Table S2: Demographic and clinical characteristics of the validation cohort; Table S3: Validation cohort performance metrics of supervised machine learning classifiers for predicting self-reported mental health outcomes from microbial features. ancombc2_results.xlsx: File containing all differentially abundant taxa by symptom.

## Abbreviations

The following abbreviations are used in this manuscript:

RDA	Recommended Dietary Allowance
BMI	Body mass index
ANCOM-BC	Analysis of Compositions of Microbiomes with Bias Correction
SCFA	Short-chain fatty acids
PCR	Polymerase chain reaction
QIIME2	Quantitative Insights Into Microbial Ecology 2 (bioinformatics platform)
GABA	Gamma-aminobutyric acid
ASA24	Automated Self-Administered 24 h Dietary Assessment Tool
Kcal	Kilocalorie(s) (unit of energy)
16S	16S ribosomal RNA gene (bacterial marker for amplicon sequencing)
ASV	Amplicon sequence variant
PCoA	Principal coordinate analysis
PERMANOVA	Permutational multivariate analysis of variance
RFC	Random Forest classifier
SD	Standard deviation
IL-8	Interleukin-8
AUC	Area under the curve
LogFC	Logarithmic fold change
FDR	False discovery rate
