# Supplementary material for: Distinct Gut Microbiome Characteristics Associated with Mental Health Symptoms of Healthy Adults
Source: Brain Sci. 2026 Mar 31;16(4):382. doi: 10.3390/brainsci16040382 (PMC13114572; doi:10.3390/brainsci16040382)
Supplement: Supplementary file 1 [file brainsci-16-00382-s001.zip › brainsci-4154086-supplementary.pdf]

Supplemental figures:

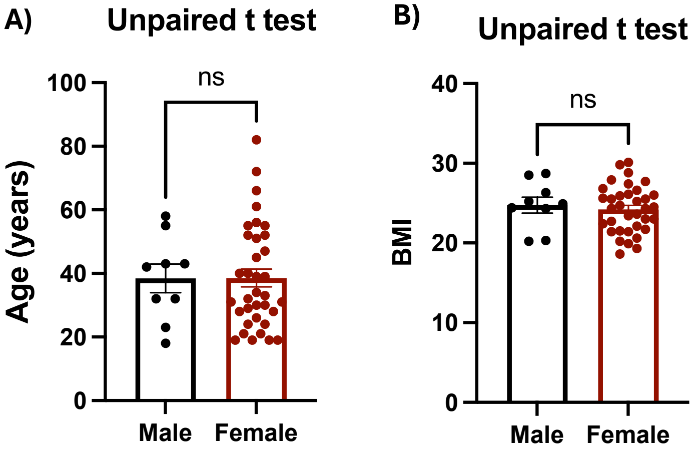

Figure S1. No Significant differences between sexes in age and BMI. Unpaired t-test (two-tailed) analysis was performed with Welch's correction to compare A) age and B) BMI between males and females.

Symptom Group	Stress	Anxiety	Depression	Sleep problems
Mean Accuracy (±SD)	0.884 ± 0.083	0.607 ± 0.138	0.718 ± 0.179	0.780 ± 0.154
Mean Balanced Accuracy (±SD)	0.668 ± 0.217	0.497 ± 0.105	0.516 ± 0.053	0.600 ± 0.221
Mean Specificity(±SD)	0.970 ± 0.054	0.845 ± 0.196	0.919 ± 0.114	0.905 ± 0.135
Mean Sensitivity (±SD)	0.208 ± 0.315	0.150 ± 0.207	0.113 ± 0.218	0.125 ± 0.250
Mean F1 weighted (±SD)	0.854 ± 0.111	0.547 ± 0.155	0.657 ± 0.200	0.749 ± 0.190
Mean ROC-AUC (±SD)	0.663 ± 0.123	0.416 ± 0.149	0.400 ± 0.188	0.673 ± 0.163

Table S1. Random forest classification performance using participant-level grouped cross-validation. All cross-validation folds were constructed at the participant level (GroupKFold), ensuring no participant contributed samples to both training and test sets within any fold. Classification performance was assessed using the mean (± SD) accuracy, balanced accuracy, specificity, sensitivity, F1 weighted, and area under the receiver operating characteristic curve (AUC), across folds.

Variable	
**Sex**	**n (%)**
Male	38 (35%)
Female	70 (65%)
**BMI, Mean (SD)**	28.5 (5.94)
**Race**	**n (%)**
White	81 (75%)
Black	11 (10.2%)
Hispanic	12 (11.1%)
American Native	1 (0.01%)
Asian	3 (2.8%)
**Mental Health Symptoms**	**n (%)**
Stress	4 (4%)
Depression	20 (19%)
Anxiety	29 (27%)
Sleep Problems	16 (15%)

Table S2. Demographic and clinical characteristics of the validation cohort. The cohort is composed of 108 participants. In contrast to the study cohort, the validation cohort includes participants with chronic diseases as well as obese individuals.

Variable	Stress	Anxiety	Depression	Sleep problems
Accuracy	0.75	0.731	0.815	0.852
Balanced Accuracy	0.510	0.5	0.5	0.5
Sensitivity	0.30	0	0	0
Specificity	0.769	1	1	1
F1 weighted	0.826	0.618	0.732	0.784
ROC-AUC	0.534	0.601	0.586	0.526

Table S3. Validation Cohort Performance metrics of supervised machine learning classifiers for predicting self-reported mental health outcomes from microbial features. Independent samples: 108; Independent subjects: 108; Features used: 1083; Missing independent features filled with zero: 365; n_extra_independent_features_ignored: 321.
